# Influence of Ionomer Content in the Catalytic Layer of MEAs Based on Aquivion^®^ Ionomer

**DOI:** 10.3390/polym13213832

**Published:** 2021-11-05

**Authors:** Irene Gatto, Ada Saccà, David Sebastián, Vincenzo Baglio, Antonino Salvatore Aricò, Claudio Oldani, Luca Merlo, Alessandra Carbone

**Affiliations:** 1 CNR-ITAE, Institute for Advanced Energy Technologies “N. Giordano”, Via Salita S. Lucia sopra Contesse 5, 98125 Messina, Italy; ada.sacca@itae.cnr.it (A.S.); vincenzo.baglio@itae.cnr.it (V.B.); antonino.arico@itae.cnr.it (A.S.A.); alessandra.carbone@itae.cnr.it (A.C.); 2 Instituto de Carboquímica, CSIC, Miguel Luesma Castán 4, 50018 Zaragoza, Spain; dsebastian@icb.csic.es; 3 Solvay Specialty Polymers, Viale Lombardia 20, 20021 Bollate, Italy; claudio.oldani@solvay.com (C.O.); luca.merlo@solvay.com (L.M.)

**Keywords:** Aquivion^®^, short side chain, dispersing agent in catalytic ink, ionomer optimization, automotive applications, PEFC

## Abstract

Perfluorinated sulfonic acid (PFSA) polymers such as Nafion^®^ are widely used for both electrolyte membranes and ionomers in the catalytic layer of membrane-electrode assemblies (MEAs) because of their high protonic conductivity, σ_H_, as well as chemical and thermal stability. The use of PFSA polymers with shorter side chains and lower equivalent weight (EW) than Nafion^®^, such as Aquivion^®^ PFSA ionomers, is a valid approach to improve fuel cell performance and stability under drastic operative conditions such as those related to automotive applications. In this context, it is necessary to optimize the composition of the catalytic ink, according to the different ionomer characteristics. In this work, the influence of the ionomer amount in the catalytic layer was studied, considering the dispersing agent used to prepare the electrode (water or ethanol). Electrochemical studies were carried out in a single cell in the presence of H_2_-air, at intermediate temperatures (80–95 °C), low pressure, and reduced humidity ((50% RH). %). The best fuel cell performance was found for 26 wt.% Aquivion^®^ at the electrodes using ethanol for the ink preparation, associated to a maximum catalyst utilization.

## 1. Introduction

Polymer electrolyte membrane fuel cells (PEMFCs) have been developed as an efficient and eco-friendly energy-conversion device for distributed power generation, transportation, and stationary uses [1,2]. Notwithstanding the advances in PEMFC technology, durability and cost remain the two main challenges to be overcome for their commercialization [3,4,5]. Durability and cost are strictly related, since they mainly depend on the perfluorosulfonic acid (PSFA) membrane and Pt-Group-Metal (PGM) catalysts used as core components of the PEMFC [6,7,8]. In particular, the Pt loading reduction is often associated to a reduction in durability; thus, the current direction is the development of membrane-electrode assemblies (MEAs) with a low Pt loading and long lifetime. In order to reach this target, it is necessary to develop MEAs with highly efficient catalytic layers (CLs). While, the effect of MEA manufacturing processes on cell performance has been investigated by different authors [9,10,11,12,13], minor attention has been devoted to the role of ionomers in the CL. The ionomer can influence the performance of fuel cells, and in fact it affects the ionic conductivity, the catalyst utilization, and the mass transport of the catalytic layer [14,15,16,17]. 

The most used ionomer is Nafion^®^, a long-side-chain perfluorosulfonic acid, considered as the benchmark for several kinds of applications ranging from energy production to sensors. A wide literature is available about the use of Nafion^®^ in both membranes and catalytic ink preparation reporting the use of the pristine polymer or composite with inorganic and organic materials [18,19,20,21]. With the aim of overcoming some limitations of Nafion^®^ when it operates in more drastic conditions, such as high temperature and low relative humidity, a short-side-chain ionomer can be used. In particular, Aquivion^®^, developed by Solvay Specialty Polymers, is a short-side-chain (SSC) perfluorosulfonic polymer and presents a higher crystallinity, higher glass-transition temperature (T_g_) and lower swelling than Nafion^®^ together with a high proton conductivity, making this SSC polymer more suitable to work in a PEMFC at higher cell temperature and lower relative humidity levels (RH, %) [22,23,24,25,26]. Talukdar et al. observed a correlation between the composition of the ionomer and the durability, with SSC polymer being chemically less stable than long-side-chain polymer [27]. Instead, Shahgaldi et al. found a better compatibility of SSC ionomers in the catalytic layer compared to their long-side-chain counterparts [28]. It is thus relevant that the ionomer structure plays an important role in cell performance and durability aspects.

The use of different ionomers requires an optimization of the entire composition of the catalytic ink, in terms of ionomer amount, dispersing agent, and thermal treatment [29,30,31]. Garsany et al. investigated the effects of the fuel cell component preparation method with SSC ionomers by comparing Aquivion^®^ with Nafion^®^, and finding superior fuel cell power density upon appropriate catalyst-loading optimization, with durability issues still a remaining challenge [32,33]. In our previous and recent study [34], it was found that the Aquivion^®^-polymer-based MEAs, treated at 125 °C, although presenting the highest performance loss after an accelerated stress test (AST), maintains a lower degradation in terms of H_2_ crossover, electrochemical surface area (ECSA), and double layer capacitance (C_dl_) between the beginning (BoT) and the end of test (EoT). 

In this work, an investigation of the influence of the dispersing agent and ionomer content in the catalytic ink on fuel cell performance was carried out. Different gas diffusion electrodes (GDEs) with different dispersing agents and different ionomer amounts were prepared and coupled with Aquivion^®^ membranes. The obtained MEAs were electrochemically characterized in a 25 cm^2^ single cell in the presence of H_2_-air, at intermediate temperatures (80–95 °C), low pressure, and reduced humidity (50% RH), to evaluate how the catalytic ink composition influenced the electrochemical performance.

## 2. Materials and Methods

### 2.1. Materials

#### 2.1.1. Electrocatalyst Preparation

The cathode catalyst consisted of a 50 wt.% PtCo, supported on Ketjenblack EC (PtCo/KB) with a nominal alloy composition of Pt_3_Co_1_ (at.), prepared by an incipient wetness of cobalt nitrate on an amorphous PtOx/C catalyst, as reported elsewhere [35]. The anode catalyst was a 50 wt.% Pt/KB. This catalyst was prepared according to a previous work [36].

#### 2.1.2. Membrane and Ionomer Preparation

The membrane used in this work was an experimental Aquivion^®^-reinforced membrane developed by Solvay Specialty Polymers and named R79-01SX^+^. The membrane (10 μm in thickness) was prepared by ePTFE web impregnation starting from the commercially available Aquivion^®^ PFSA D79-25BS water-based dispersion. The membrane-manufacturing process was reported elsewhere [37]. Regarding the ionomer, a dispersion based on Aquivion^®^ PFSA, D79-20BS with an EW: 790 g mol^−1^, and water as a dispersing solvent was used in the catalytic layer.

#### 2.1.3. Electrode and MEA Preparation

In-house prepared electrodes were realized by the spray-coating technique [38,39,40,41]. The catalytic ink was obtained by mixing the developed catalysts and Aquivion^®^ ionomer dispersions D79-20BS at different percentages, ranging between 16 and 36 wt.%. Moreover, H_2_O or ethanol (EtOH) was used as a dispersing agent to prepare the catalytic ink. A 20 wt.% of ammonium carbonate (Carlo Erba Reagents s.r.l., Milano Italy) was used as a pore-former. The catalytic ink was deposited onto the commercial gas diffusion layer Sigracet-25BC (SGL group). A Pt loading of 0.2 mg cm^−2^ both for the anode and cathode side was used.

Membrane-electrode assemblies (MEAs) were obtained by hot pressing the electrodes onto the R79-01SX^+^ membrane at 125 °C [34] with a pressure of 20 kg cm^−2^ for 5 min. The membranes were used as received without any further treatments.

In Table 1, a summary of the developed MEAs is reported.

### 2.2. Methods

#### 2.2.1. Electrocatalysts Characterization

The electrocatalyst powders were characterized to confirm the morphology, the crystallographic structure, and the degree of alloying. Transmission Electron Microscopy (TEM) was carried out through a Philips (Amsterdam, NL) CM12 microscope by depositing the catalyst powder dispersed in 2-propanol on a carbon-film-coated Cu grid.

X-ray diffraction (XRD) analysis was performed using a Philips (Amsterdam, NL) Xpert 3710 X-ray diffractometer with Cu Kα radiation operating at 40 kV and 20 mA. The Pt and Pt alloy crystallite sizes were calculated from the (220) reflection in the face-centered cubic (FCC) structure, since this reflection has no other interferences with other signals, considering the Debye-Scherrer equation, and removing the contribution from the instrumental broadening.

#### 2.2.2. Membrane Characterization

The membranes were morphologically characterized using the FE-SEM Jeol JMS 7610F. The membrane cross-section was obtained through the liquid nitrogen freezing method. Sample was frozen in liquid nitrogen and then fractured, bending it. 

#### 2.2.3. Electrochemical Tests

Electrochemical tests were performed in a 25 cm^2^ single cell, at 80 °C and 95 °C, at 1.5–2 bar_abs_, and 50 RH%, by feeding the cell with H_2_ at the anode and air or O_2_ as an oxidant at the cathode. The flow rates were fixed at 2 and 1.5 times the stoichiometric value for oxidant and fuel, respectively. The single-cell performance in terms of polarization curve was investigated by steady-state galvanostatic measurements in H_2_/air. Measurements in O_2_ were carried out to determine the mass activity (j_m_) of the electrocatalysts. The cell was connected to a Fuel Cell Technologies test station including an HP6051A electronic load. An AUTOLAB Potentiostat/Galvanostat (Metrohm Autolab B.V., Netherlands), equipped with a 20 A current booster, was used for electrochemical diagnostics in terms of cyclic voltammetry (CV). CV studies, under PEMFC configuration, were carried out by feeding hydrogen to the anode that operated as both counter and reference electrode and nitrogen to the cathode (working electrode) at 1 bar_abs_. The potential ranged between 20 and 1250 mV with a sweep rate of 50 mV s^−1^. The Electrochemical Surface Area (ECSA) was determined by integrating the CV profile in the hydrogen adsorption region after a correction for the double layer capacitance. Data were not corrected for hydrogen cross-over.

## 3. Results and Discussion

**Figure 1:**
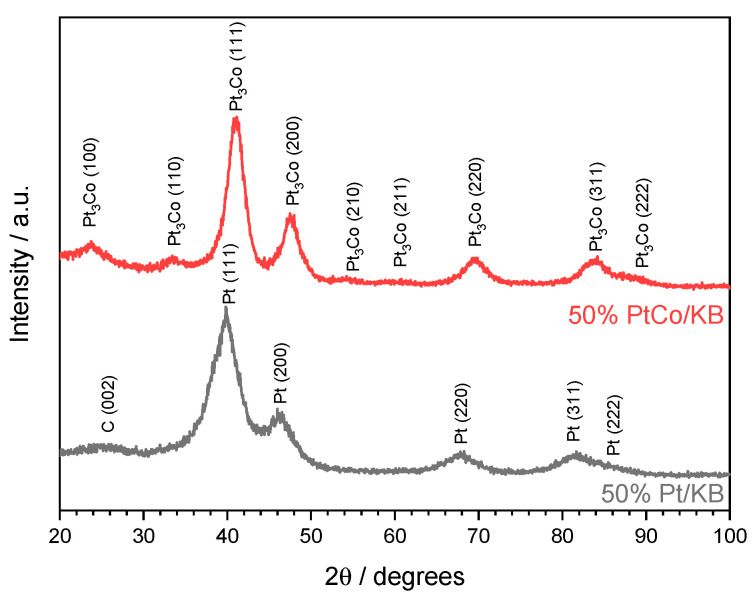
XRD patterns for Pt/KB (lower, black) and PtCo/KB (upper, red) electrocatalysts.

The XRD patterns for the developed electrocatalysts (Pt/KB and PtCo/KB) are reported in Figure 1. Of note is the FCC structure of Pt for Pt/KB, with a crystallite size of 2.5 nm. The PtCo/KB catalyst presents a single-ordered primitive cubic (L1_2_) phase for the alloy, with reflections slightly shifted towards larger Bragg angles in comparison to Pt/KB. In this case, a crystallite size of 3.3 nm was calculated [35].

**Figure 2:**
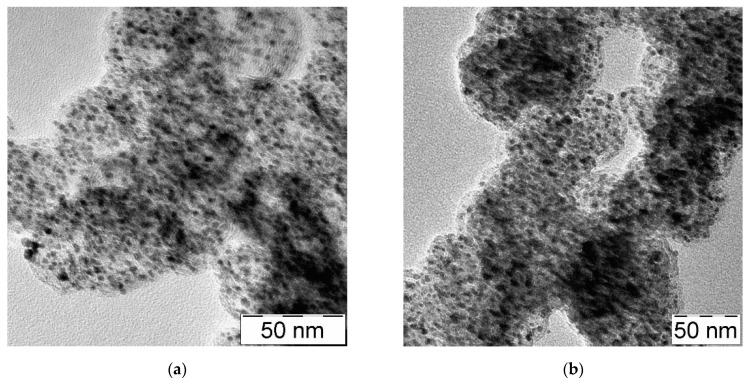

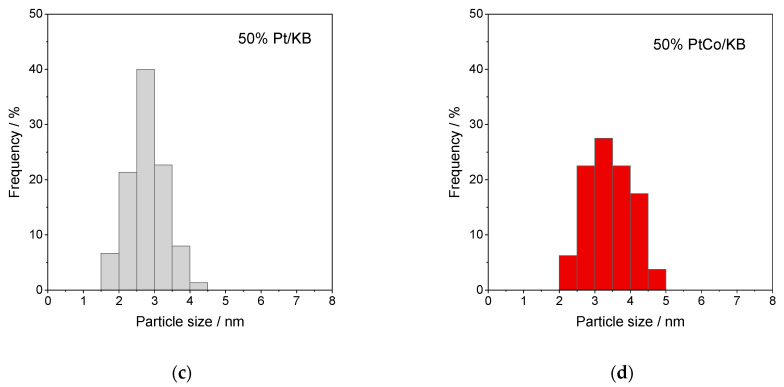
TEM images and particle-size distribution for Pt/KB (**a**,**c**) and PtCo/KB (**b**,**d**).

TEM images highlight a good dispersion of the Pt particles (Figure 2a) and the metallic PtCo (Figure 2b) alloy on the carbonaceous support. Moreover, the particle size derived from TEM images of 2.8 nm for Pt/KB (Figure 2c) and 3.4 nm for PtCo/KB (Figure 2d) confirm the data obtained from the XRD characterization.

**Figure 3:**
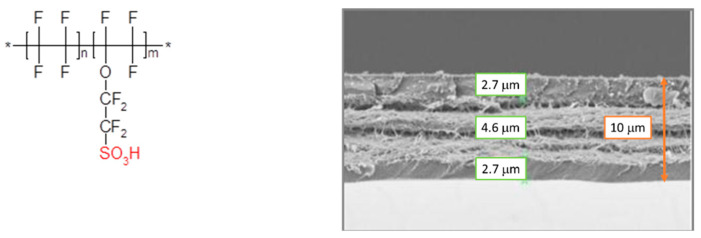
Chemical structure of Aquivion^®^ PFSA (**left**) and SEM images of cross-section of the Aquivion^®^ R79-01SX^+^ membrane (**right**).

In Figure 3, the chemical structure of the short-side-chain Aquivion^®^ PFSA polymer and the SEM image of the R79-01SX^+^-reinforced membrane are shown. The cross-section image confirms that the membrane has a total thickness of about 10 μm and it consists of three layers with a smooth surface and tight interpenetration between the support and ionomer. 

Thanks to the support, the thin membrane shows improved mechanical properties, when compared to the extruded congener, and, notwithstanding low thickness, shows a longer durability in AST than thicker ones, while maintaining a high conductivity, as reported elsewhere [31].

**Figure 4:**
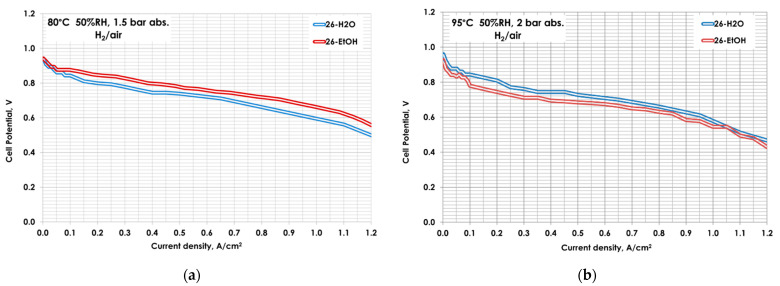
Polarization curves for 26-H_2_O and 26-EtOH at (**a**) 80 °C 50 RH% and 1.5 bar_abs_ (**b**) 95 °C 50 RH% and 2 bar_abs_.

Few studies [42,43,44,45] have been dedicated to the effects of the dispersion medium on the CL structure. A good selection of the dispersion agent is necessary to obtain the desired viscosity, surface tension, and control of the aggregate size in the CL above all when a spraying technique is used for deposition. To optimize the catalytic ink composition, the influence of dispersing agent, maintaining as a constant the ionomer loading, was firstly carried out. Starting from a previous paper, in which a correlation between the sulfonic groups and total ionomer content in the catalytic layer was found [29], an ionomer loading of 26 wt.% was calculated by considering that the EW of the Aquivion^®^ PFSA polymer used in this work is 790 g mol^−1^. Moreover, in order to evaluate the influence of the dispersing agent of the catalytic ink, initially H_2_O (26-H_2_O), and, successively, EtOH (26-EtOH) were used. Such prepared MEAs were electrochemically characterized in terms of I–V curves at different operating conditions. In Figure 4, the polarization curves are reported.

At 80 °C and 50%RH, the EtOH used as dispersing agent for the ink seems to improve the performance (Figure 4a). This phenomenon could be explained considering that alcohol is able to swell the ionomer, making the sulfonic groups more accessible, producing a more homogeneous distribution of the ionomer in the catalytic layer [30], in particular at a reduced gas humidification. At 95 °C, the trend seems to be different. In fact at a low current density, the performance of MEA 26-H_2_O is slightly higher than the 26-EtOH, whereas at practical cell potential (0.6–0.7 V), the curves are almost the same: EtOH-based electrode preparation produces a similar performance than that prepared using water (Figure 4b). 

As known, the I–V curves in H_2_/air provide information on the whole MEA, including various effects such as: membrane, diffusive layer, water-management phenomena, etc. With the aim of better understanding the effect of the dispersing agent on the catalytic layer and its interaction with the catalyst, the most indicative electrochemical parameters, ECSA and j_m_, were considered.

In Table 2, j_m_ (calculated in O_2_) and the ECSA for both MEAs in two different operating conditions are reported. The improvement of the ionomer dispersion in the catalytic layer, promoted by the addition of EtOH in the ink, is more evident in the activation zone, wherein the MEA prepared with EtOH achieves a better j_m_. This is well in agreement with an increase in ECSA.

A higher functional-group availability of the ionomer dispersion in the catalyst layer positively influences three-phase boundary formation and, consequently, the platinum utilization. Accordingly, EtOH was selected for the catalytic ink preparation.

**Figure 5:**
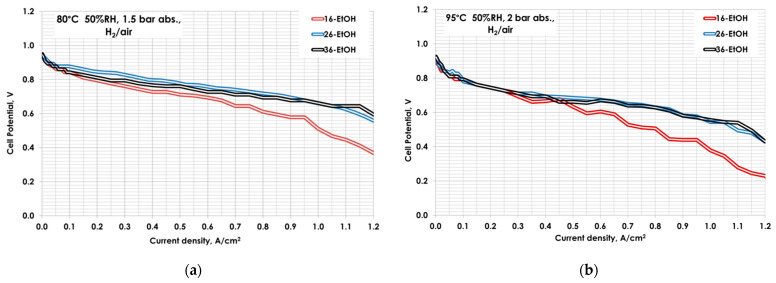
Polarization curves for 16- EtOH, 26-EtOH, and 36-EtOH at (**a**) 80 °C 50 RH% and 1.5 bar_abs_ (**b**) 95 °C 50 RH% and 2 bar_abs_.

In order to validate the correlation reported in reference [29] also with the Aquivion^®^ ionomer and to identify the optimal catalytic ink composition, three different ionomer contents ranging from 16 to 36 wt.% were evaluated using EtOH as a dispersing agent. Electrochemical characterizations in single cells, in terms of I–V curves at 80 °C and 95 °C, 50% RH, and low pressure are reported in Figure 5.

For both operating temperatures, the amount of 16 wt.% shows the worst performance, probably due to a poor electronic and ionic percolation within the catalytic layer.

The increase in the ionomer amounts up to a 26 wt.% produces an evident benefit on the fuel cell performance, especially at a higher current density. A further increase up to 36 wt.% of ionomer maintains unaltered the performance. This trend is the same both at 80 °C and 95 °C. In particular, in operating conditions typical for automotive applications (95 °C), current densities of 600 mA cm^−2^, 870 mA cm^−2^ and 850 mA cm^−2^ at 0.6V were recorded for the 16-EtOH, 26-EtOH, and 36-EtOH MEAs, respectively.

In addition, this behavior is also confirmed by an analysis of the electro-kinetic parameters reported in Table 3. In different operating conditions, the best electrochemical surface area (ECSA) and j_m_ values were recorded relatively to the 26 wt.% ionomer loading, demonstrating that optimal characteristics of ionic/electronic percolation and interface are obtained using this content.

**Figure 6:**
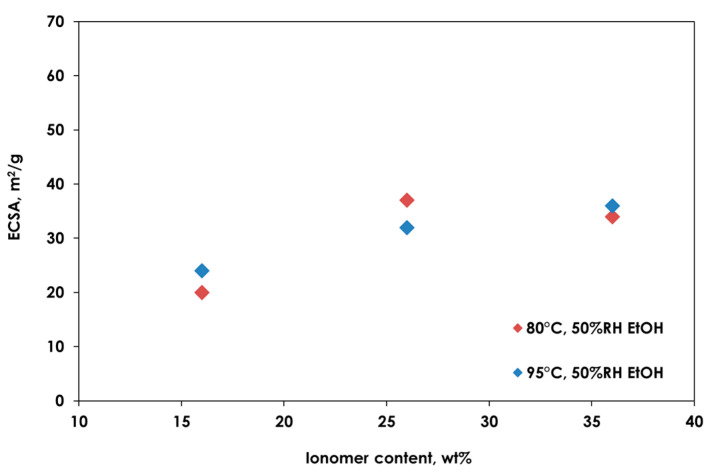
ECSA variation as a function of the different ionomer amounts and different operating conditions.

In Figure 6, the ECSA variation as a function of the different ionomer amounts and different operating conditions is shown.

It is evident that the ECSA of the MEA with the 26 wt.% of ionomer loading is the highest for all operating conditions investigated.

As found for long-side-chain (LSC) ionomers, such as Nafion^®^, an increased ionomer content increases the three-phase reaction zone, producing a higher ECSA and improving the ionic conductivity in the catalytic layer. On the other side, a further increase in ionomer could produce an insulating effect on the catalyst particles, reducing the catalyst utilization and/or the electronic conductivity. Moreover, the increase in ionomer content produces a thickness increase in the ionomer film on the catalyst particles and a diffusion barrier for both gas and water transport [46]. In conclusion, the optimal ionomer content is strongly dependent on the type of polymer and its EW. Moreover, the dispersing agent also affects the performance and electrochemical parameters, when the same optimal ionomer loading is used.

## 4. Conclusions

The influence of the ionomer amount in the catalytic layer was studied, considering the dispersing agent used to prepare the electrode (water or ethanol). The optimal ionomer amount depends on the polymer characteristics, specific catalyst, and the operating conditions. In particular, it was found ethanol is a proper dispersing agent for the catalytic ink preparation for the Aquivion^®^ ionomer, a promising SSC polymer, since both performance and ECSA improvements were recorded compared to using water as solvent. Moreover, the electrode formulation based on a 26 wt.% of ionomer content reaches the highest performance both at 80 and 95 °C, indicating an optimized catalytic ink composition. Additionally, the MEA with a 26 wt.% of ionomer content shows the highest values for the electro-kinetic parameters, such as ECSA and j_m_, confirming a good electronic and ionic percolation within the catalytic layer. 

## Figures and Tables

**Table 1:** MEAs developed.

MEA	Catalytic Ink Dispersion Agent	Ionomer Amount, wt.%
26-H_2_O	H_2_O	26
26-EtOH	EtOH	26
16-EtOH	EtOH	16
36-EtOH	EtOH	36

**Table 2:** Electro-kinetic parameters for MEAs with different dispersing agents.

Operating Conditions	j_m_ @ 0.9V_IRfree_, mA mg^−1^		ECSA, m^2^ g^−1^	
	26-H_2_O	26-EtOH	26-H_2_O	26-EtOH
80 °C, 50%RH, 1.5 bar	257	304	36	37
95 °C, 50%RH, 2 bar	257	357	30	32

**Table 3:** Electro-kinetic parameters for developed MEAs with different ionomer amounts.

MEA	80 °C, 50%RH		95 °C, 50%RH	
	ECSA, m^2^ g^−1^	j_m_ @ 0.9V_IRfree,_ mA mg^−1^	ECSA, m^2^ g^−1^	j_m_ @ 0.9V_IRfree,_ mA mg^−1^
16-EtOH	20	207	24	106
26-EtOH	37	304	32	357
36-EtOH	34	155	36	205

## Data Availability

The data presented in this study are available on request from the corresponding author.
